# Accuracy of intraoperative estimation of femoral stem anteversion in cementless total hip arthroplasty by using a digital protractor and a spirit level

**DOI:** 10.1186/s13018-020-02183-7

**Published:** 2021-01-07

**Authors:** Anuwat Pongkunakorn, Nawakun Phetpangnga, Narawit Kananai

**Affiliations:** Department of Orthopaedic Surgery, Lampang Hospital and Medical Educational Center, Lampang, Thailand

**Keywords:** Femoral stem anteversion, cementless total hip arthroplasty, digital protractor, spirit level

## Abstract

**Background:**

The femoral component anteversion during surgery is traditionally assessed by a visual assessment of the surgeon and has proven to be imprecise. We sought to determine the accuracy of a digital protractor and a spirit level to measure the stem anteversion during cementless THA.

**Methods:**

A prospective study was conducted among 107 patients (114 hips) who underwent primary cementless THA via posterolateral approach. A pipe with a spirit level was attached to the tibial tubercle and intermalleolar midpoint. While the leg was held perpendicularly to the floor, stem anteversion was estimated by 3 methods: method A by visual assessment; method B by a digital protractor alone; and method C by a digital protractor combined with a spirit level. The angles were compared with the true anteversion measured by postoperative CT scan.

**Results:**

The average anteversion by method C (22.8° ± 6.9°, range -2° to 40°) was significantly lower than method A (24.6° ± 5.2°, range 0° to 30°) (p=0.033), but not different from the true anteversion (22.1° ± 8.2°, range -5.4° to 43.1°) (p=0.445). There were no significant differences between method B (23.2° ± 8.2°, range -4° to 45°) and method A, C or the true anteversion. The mean deviation of the intraoperative estimation from the true anteversion was 0.8° ± 3.7° (range -7.1° to 8.0°) by method C; 1.2° ± 5.1° (range -8.8° to 14.3°) by method B; and 2.5° ± 7.4° (range -19.0° to 16.0°) by method A. Estimation error within 5° was found in 107 hips (93.9%) with method C; 86 hips (75.4%) with method B; and 59 hips (51.8%) with method A.

**Conclusion:**

Accurate estimation of stem anteversion during cementless THA can be determined intraoperatively by the use of a digital protractor and a spirit level.

**Trial registration:**

Thai Clinical Trials Registry (TCTR 20180326003). Registered on 20 March 2018. Retrospectively registered.

## Introduction

Successful total hip arthroplasty (THA) depends on an accurate placement of the femoral and acetabular components [1, 2]. This accuracy would ensure mating of both components without impingement throughout the hip motion and requires a method to create the combined anteversion. Preparing the femur first has been recommended in cementless THA to allow the surgeon to adjust the cup anteversion according to stem anteversion in the relatively inflexible anatomy of the proximal femur [3]. The femoral component anteversion during the surgery is traditionally assessed by a visual estimation of the surgeon for the angle between the leg axis and the femoral stem axis after flexing the knee and placing the leg vertically. This technique has proven to be imprecise.

Wines and McNicol [2] studied the difference between the surgeons’ intraoperative assessment and the CT measurement and found a precision of 10.4° in 111 hips with a range of 25° underestimation to 30° overestimation. Dorr et al [4] found a poor correlation of the surgeon’s estimation in 109 hips and a precision of 11.3°. Some investigators used a manual goniometer to improve the precision and found a mean error of 7.3° [5], or estimation error ≥5° in 28% of the hips [6]. Some surgeons applied a digital protractor or a spirit level to aim the angles of the acetabular component and reduce outliers significantly [7–10]. There is no previous study regarding the accuracy of the cementless femoral component placement by using such devices. We sought to determine the validity of a digital protractor and a spirit level to measure the femoral stem anteversion during cementless THA.

The purposes of this study were (1) to evaluate the accuracies of intraoperative estimation of cementless femoral stem anteversion by using a digital protractor with or without a spirit level comparing with the conventional method that used visual estimation, and (2) to examine the factors that influenced the angle overestimation and underestimation within 5° of this new estimating method.

## Methods

A prospective study was conducted among the patients with hip osteoarthritis and femoral neck fractures who underwent primary cementless THA via posterolateral approach by one experienced surgeon between July 2017 and June 2019. Exclusion criteria were patients with previous ipsilateral tibial fractures, total knee arthroplasties and knee deformity with a tibio-femoral angle more than 5° varus or 15° valgus. The trial was approved by the institutional review board (Code 42/60) and registered in the Thai Clinical Trials Registry. All patients gave their written informed consent prior to inclusion.

### Surgical Techniques

**Fig. 1:**
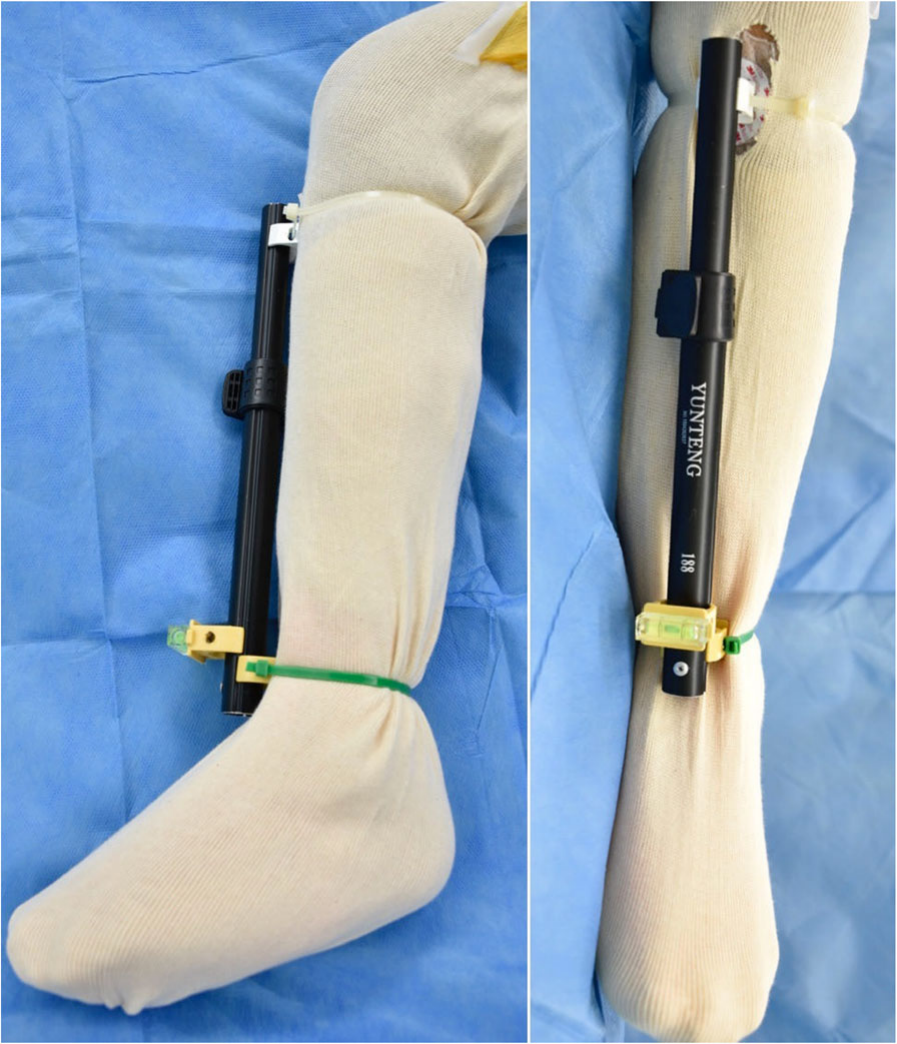
The aluminium pipe with a spirit level represented the mechanical axis of the tibia by its attachment to the intermalleolar midpoint and the medial 1/3 of the tibial tubercle

The surgery was performed via posterolateral approach. The patient was positioned in the lateral decubitus. The leg was put in a stockinette and an EKG electrode (3M Red Dot, USA) was attached to the medial 1/3 of the tibial tubercle. Two plastic pipe clips (Thai Pipe, Thailand) were attached to the anterior part of the shin with nylon cable ties. The base of one clip was positioned at the midpoint of the most medial and most lateral points of the malleoli and the other was locked onto the Red Dot electrode. An aluminium pipe (Yunteng self picture monopod YT-188, China) was gently pressed over both clips until the bilateral grooves of the pipe were snugly captured between the clip edges. This pipe would represent the mechanical axis of the tibia. A spirit level (Haccury YK-3, China) was glued to the base of another pipe clip, and then connected to the pipe by pressing the clip over the pipe (Fig. 1).

**Fig. 2:**
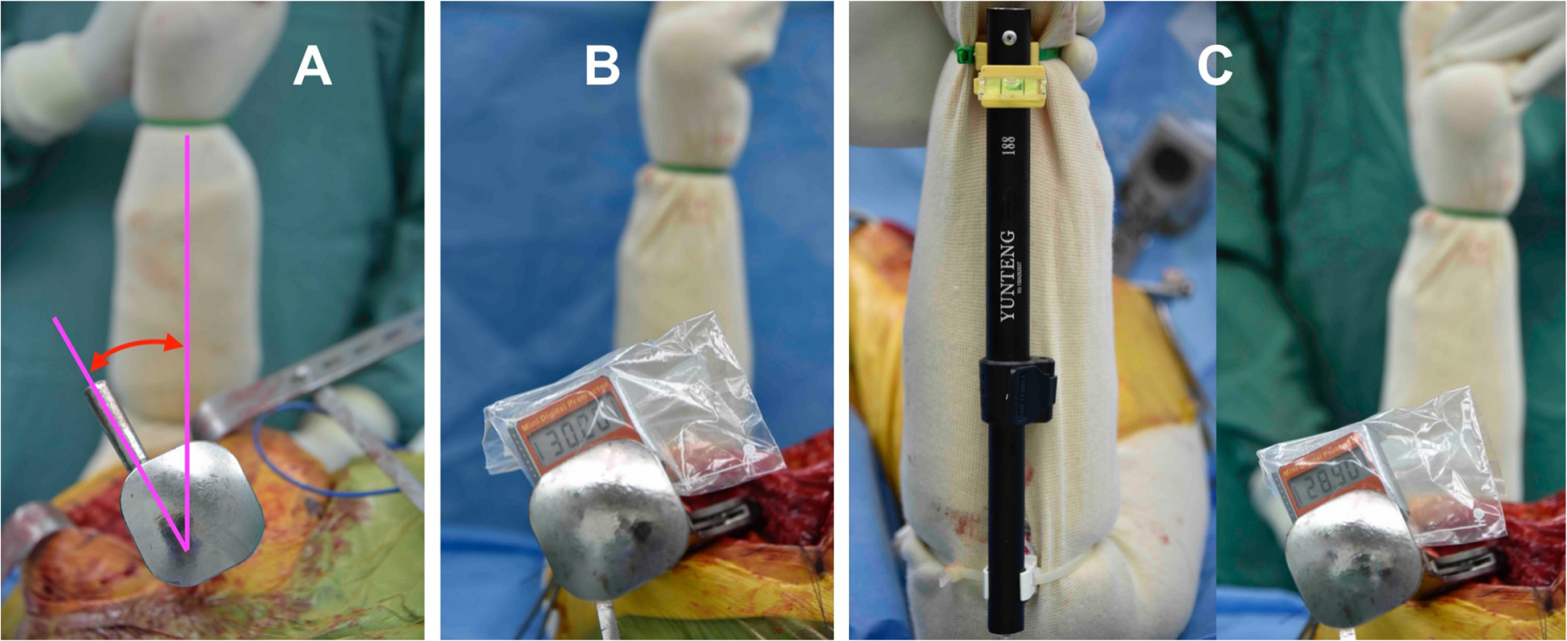
During trial stem insertion, the femur was internally rotated with knee flexion. Three methods for estimating the stem anteversion were performed sequentially. Method A by visual estimation (**a**); method B by a digital protractor alone (**b**); and method C by a digital protractor combined with a spirit level (**c**)

When the trial stem was inserted, the femur was internally rotated with knee flexion. Three methods for estimating the stem anteversion were performed sequentially. Method A by visual estimation, the surgeon assessed the angle between the leg axis and the axis of metal rod of the stem inserter handle by eye (Fig 2a). Method B by digital protractor alone, the assistant placed the leg vertically until the surgeon approved its position by visualization, without any concern to the spirit level. A digital protractor (Etopoo DC18, China) was placed on the flat surface of the stem handle and recorded as an estimated stem anteversion (Fig 2b). Method C by digital protractor and spirit level, the assistant internally rotated the leg until a bubble in the spirit level was centered. The stem anteversion was then measured by placing a digital protractor on the handle (Fig 2c).

Demographic data included patient age, gender, body mass index (BMI), diagnosis and stem type. The intraoperative estimation of stem anteversion angles by method A, B and C were recorded. All patients received CT scans postoperatively at 4–5 days, in supine position. The scans were obtained from the acetabulum to the proximal tibia with a 1.5-mm thickness using Philips Ingenuity Core 128 (Cleveland, USA). True stem version was defined as the angle between a line through the center of the neck of the femoral prosthesis and the posterior condylar line [2]. The knee alignment was measured as tibio-femoral angle in the scout view. Two intramedullary midpoints were marked at a 10-cm distance from the knee joint surfaces, one at the distal shaft of the femur and the other at the proximal shaft of the tibia. The angle between the lines drawn from the center of the bases of the tibial spines to both midpoints was defined as a tibio-femoral angle [11].

All radiographic assessments were independently performed by 2 orthopaedic residents, who were not involved with the surgery and repeated in a blind manner 4 weeks later. The average of 4 measurements was used for data analysis. Inter-observer and intra-observer measurement reliabilities were determined with intra-class correlation coefficients (ICC) using the absolute agreement and 2-way random-effects model. ICC values <0.5 indicated poor reliability, 0.5–0.74 moderate, 0.75–0.9 good, and >0.90 indicated excellent reliability [12].

The sample size was calculated to detect a significant difference in percentages of hips with intraoperative estimation error within 5°. According to the results in 25 hips of our pilot study using the traditionally visual estimation technique, 52% (13 hips) had an estimation error within 5°. We hypothesized that our method could achieve this goal in 90% of hips. With a two-sided type I error level of 0.05 and a 90% statistical power of detection in a two-dependent proportions formula, the sample size was 100 hips.

The primary outcome was the percentage of stem placements with an error within 5°. The secondary outcome was the deviation degree of the estimated stem anteversion from the true stem anteversion. The Shapiro-Wilk test for normal distribution was used prior to further statistical analysis. Continuous data were analyzed by using the t-test and Mann-Whitney U test. Categorized data were analyzed by using the exact probability test. Correlation between the estimated and true anteversion was analyzed by the Pearson correlation coefficient. Angle overestimation and underestimation were defined when the estimated anteversion was above and below the CT measurement by more than 1° respectively. We evaluated the factors that influenced the angle overestimation and underestimation in method C by using multivariate regression analysis. The statistical analyses were performed using STATA version 10.1 (Stata Corp LP, College Station, Texas, USA) and a p-value of <0.05 was considered significant.

## Results

There were 110 patients (117 hips) enrolled in the study. Two hips were excluded due to severe varus knee deformity and one hip had previous tibial fracture. There were 64 men (68 hips) and 43 women (46 hips) enrolled in the final analysis. The mean age was 56.8 ± 10.1 years (range, 25–80). Most of the diagnoses were osteonecrosis of the femoral head (53 hips, 46.5%), primary osteoarthritis (21 hips, 18.4%) and femoral neck fracture (20 hips, 17.5%). Avenir stems (Zimmer Biomet, Warsaw, Indiana, USA) were implanted in 48 hips (42.1%), Excia stems and Metha stems (Aesculap, Tuttlingen, Germany) in 57 hips (50.0%) and 9 hips (7.9%), respectively (Table 1).

**Table 1:** Patients’ baseline characteristics (n=114 hips).

Data	Number (%)
**Age** (year)	
mean (SD)	56.8 (10.1)
range	25–80
**Gender** male : female	64 : 43
**Body mass index** (kg/m^2^)	
mean (SD)	22.7 (3.7)
range	15.8–35.0
**Diagnosis:**	
Osteonecrosis of femoral head	53 (46.5%)
Primary osteoarthritis	21 (18.4%)
Femoral neck fracture	20 (17.5%)
Hip dysplasia	12 (10.6%)
Post-traumatic osteoarthritis	5 (4.4%)
Inflammatory joint disease	3 (2.6%)
**Stem type** N (%)	
Excia (Aesculap)	57 (50.0%)
Avenir (Zimmer-Biomet)	48 (42.1%)
Metha (Aesculap)	9 (7.9%)
**Tibio-femoral angle** ^a^	
mean (SD)	5.1° (3.2°)
range	-2.9° to 11.9°

^a^ a positive value represents valgus, and a negative represents varus alignment

The average stem anteversion by method A was 24.6° ± 5.2° (range, 0° to 30°); by method B was 23.2° ± 8.2° (range, -4° to 45°); by method C was 22.8° ± 6.9° (range, -2° to 40°) and the true anteversion angle was 22.1° ± 8.2° (range, -5.4° to 43.1°). Angles by method A were significantly higher than by method C (p=0.033) and the true anteversion (p=0.006).

**Fig. 3:**
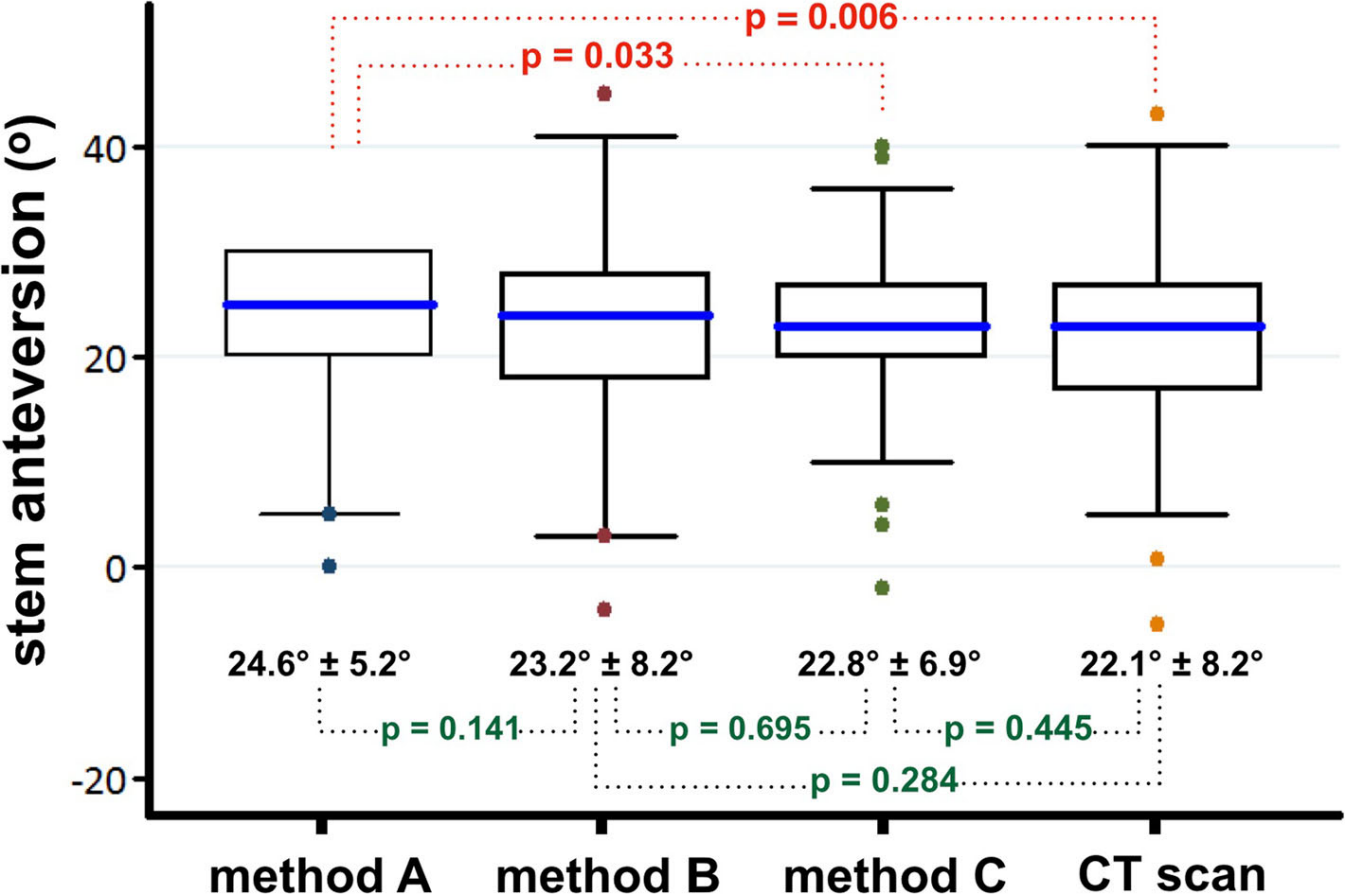
Anteversion angles of femoral component, comparing between the method **a**, **b**, **c** and CT scan. Boundaries of the boxes, 25^th^ and 75^th^ percentiles; horizontal lines inside the boxes, median; whiskers and large dots, maximum and minimum; dotted line, statistical comparison between mean values of the paired methods

Interestingly, the estimated angle by a digital protractor combined with a spirit level was not significantly different from the true anteversion angle (p=0.445). Likewise, non-significant differences were demonstrated when compared between the stem anteversion by a digital protractor alone and those by visual assessment (p=0.141); by a digital protractor combined with a spirit level (p=0.695); as well as the true anteversion angle (p=0.284) (Fig 3).

**Fig. 4:**
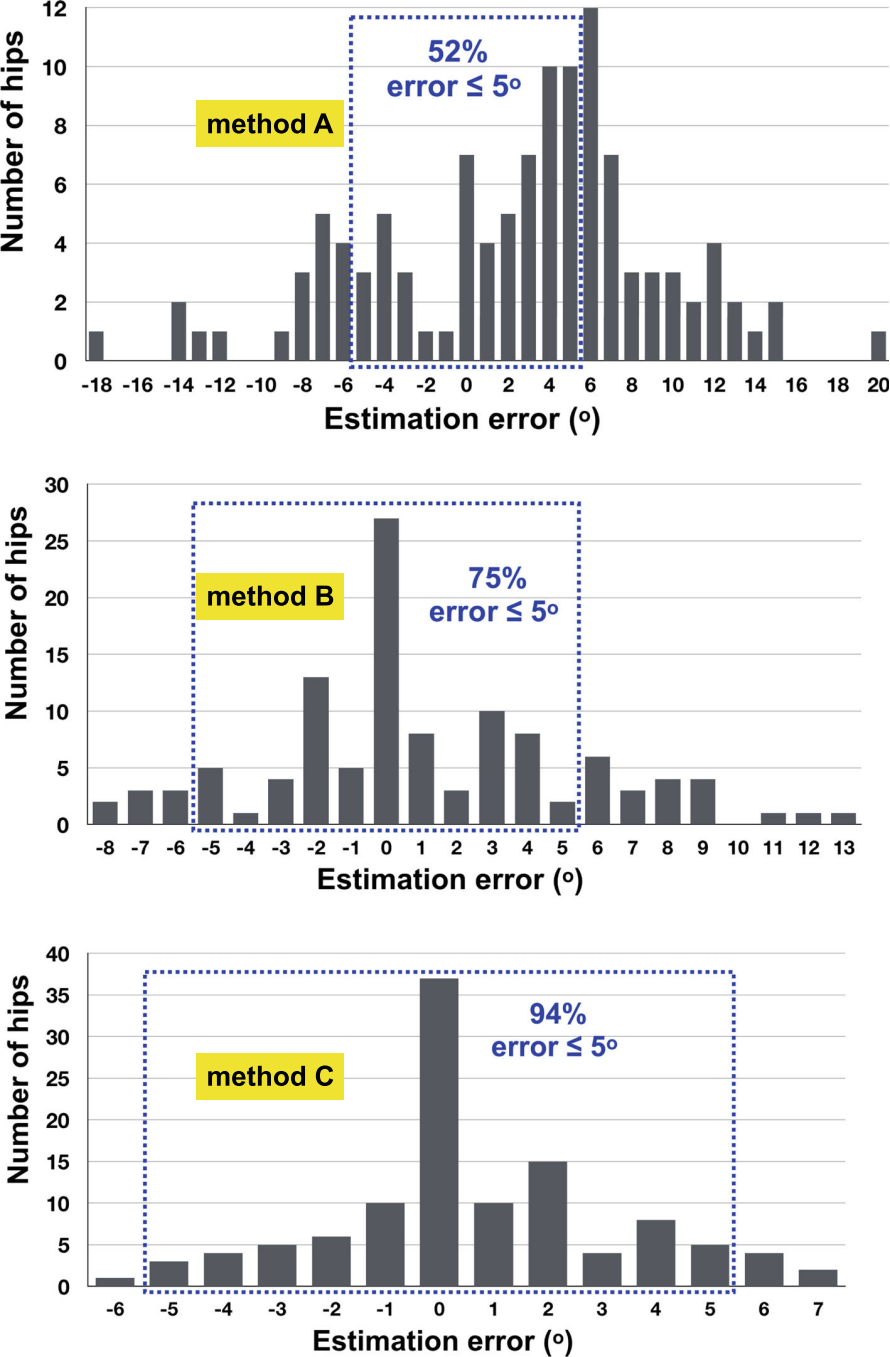
Distribution of errors in intraoperative estimation from the true anteversion by CT scan of the method **a**, **b** and **c**. The dotted zone represents the percentage of stems with errors within 5°. Method A 52%; method B 75 %; and method C 94%

The mean deviation of the intraoperatively estimated anteversion from the true anteversion was 0.8° ± 3.7° (95% CI 0.1° to 1.5°, range -7.1° to 8.0°) by method C; 1.2° ± 5.1° (95% CI 0.2° to 2.1°, range -8.8° to 14.3°) by method B; and 2.5° ± 7.4° (95% CI 1.1° to 3.9°, range -19.0° to 16.0°) by method A. Estimation error within 5° was found in 86 hips (75.4%) with using digital protractor alone; significantly lower than 93.9% (107 hips) by using both digital protractor and spirit level (p<0.001); but significantly higher than 51.8% (59 hips) by visual assessment (p<0.001) (Fig 4). In other words, estimation error >5° was most commonly found in the visual assessment method. This risk could be minimized by a digital protractor with or without a spirit level. With digital protractor and spirit level method, the risk difference was -0.42 (95% CI -0.52 to -0.32) and the risk ratio was 0.13 (95% CI 0.06 to 0.27) (p<0.001). With digital protractor alone, the risk difference was -0.24 (95% CI -0.36 to -0.12) and the risk ratio was 0.51 (95% CI 0.35 to 0.74) (p<0.001).

**Fig. 5:**
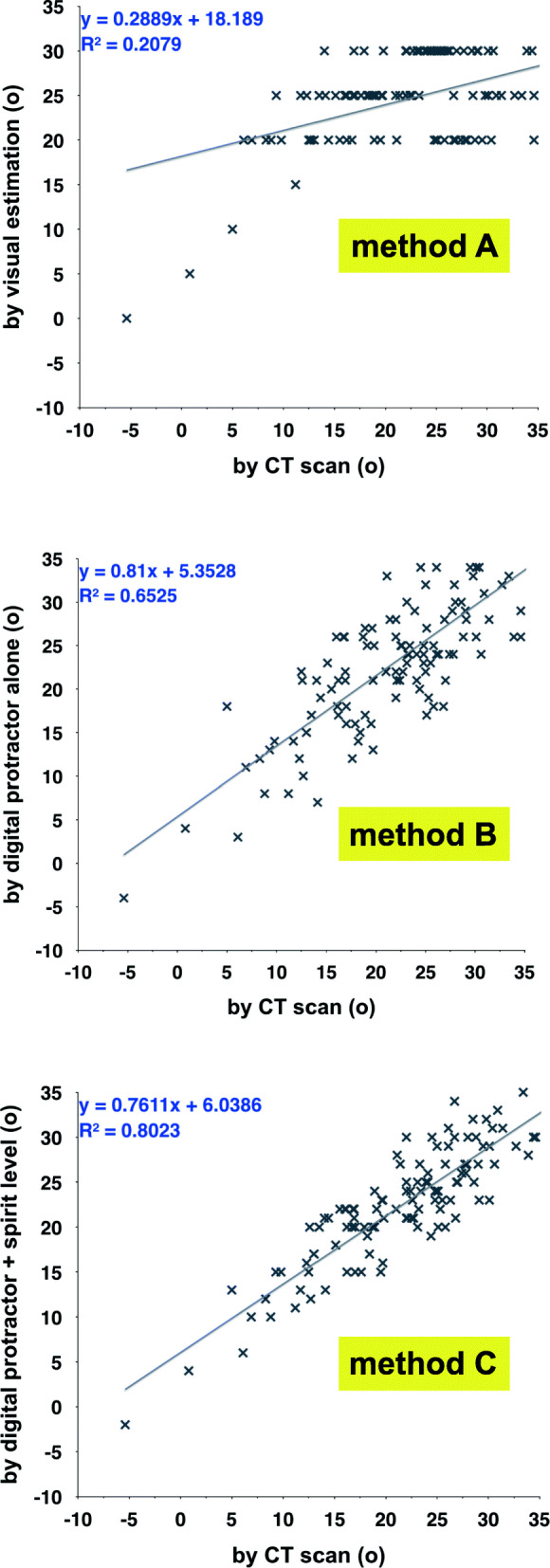
Intraoperative estimation of stem anteversion compared with CT scan shows a low positive correlation in method **a** (r = 0.456), but high positive correlations in method **b** (r = 0.808) and method **c** (r = 0.896). A solid line represents the linear regression between the estimated and true anteversion (r^2^)

The Pearson correlation coefficient of the true anteversion and the estimated anteversion was 0.456 for method A; 0.808 for method B; and 0.896 for method C (Fig 5) with a significant correlation between the measurements (p<0.001). The ICC for intra- and inter-observer reliability of anteversion measurements were 0.98 and 0.98, respectively. The ICC for intra- and inter-observer reliability of tibio-femoral angle measurements were 0.91 and 0.73, respectively. Among the factors analyzed using regression analysis in method C, only the tibio-femoral angle significantly influenced the angle overestimation within 5° (p=0.008) (Table 2). No factors were identified to influence angle estimation in hips with underestimation. Hips with an error within 5° had the average tibio-femoral angle of 4.5° ± 2.6°. Hips with an overestimation >5° and underestimation >5° had the mean tibio-femoral angle of 7.9° ± 3.1° (p<0.001) and 2.4° ± 3.4° (p=0.072), respectively. There was one dislocation but no surgical site infection postoperatively. The average duration of follow-up was 27 ± 7 months (range, 16 to 39).

**Table 2:** Factors influencing the angle overestimation and underestimation within 5° in method C, analyzed by using multivariate regression.

Factors	Overestimation within 5° (n=42 hips) p-value	Underestimation within 5° (n=28 hips) p-value
**Age**	0.460	0.843
**Sex**	0.925	0.893
**Body mass index**	0.773	0.984
**Diagnosis**	0.652	0.955
**Stem type**	0.776	0.866
**True anteversion**	0.549	0.671
**Tibio-femoral angle**	**0.008**	0.164

## Discussion

Currently, computer navigation is the most accurate method to measure the femoral stem anteversion intraoperatively with a precision of 5° and no outliers beyond 6° [3]. Interestingly, other digital devices have not been utilized to improve this estimation error during cementless femoral prosthetic implantation. We investigated whether using a digital protractor and a spirit level provided the technical ability for the surgeon to assess the anteversion of the cementless stem intraoperatively with the target of 5°estimation error.

Assessment of the femoral component anteversion during the surgery by visual estimation found in this study had a precision of 7.4° with a range of 19° underestimation to 16° overestimation. Estimation error within 5° was found in only 52% of hips. Concordance with the previous studies that found the precision of 10.4°–11.3° [2, 4]. Placing a digital protractor on the trial stem handle could improve the precision to be 5.1° with a range of 9° underestimation to 14° overestimation, and 75% of hips had an estimation error within 5°. The risk of estimation error >5° was reduced by 49% and the absolute difference was -24%. Using this technique in an estimated 4 THAs would prevent 1 unacceptable estimation error.

The best outcomes belonged to the method that used both digital protractor and spirit level with a precision of 3.7°. The mean absolute value of error was 0.8°, range from 7° underestimation to 8° overestimation, and 94% of stems had an error within 5°. The risk of estimation error >5° was significantly reduced by 87% and the absolute difference was -42%. Using this method in an estimated 2 THAs would prevent 1 unacceptable estimation error. Correlation coefficient between the estimated angle by these devices and the true anteversion by CT scan revealed a high positive correlation [13]. This significant benefit affirmed the same findings of our previous clinical trial in 31 patients with femoral neck fractures who underwent cemented bipolar hemiarthroplasty [14]. We found this method could improve surgeons’ estimation of cemented stem anteversion with the mean absolute error of -0.2° (SD 3.0°, range -5.4° to 7.0°) and 28 stems (90.3%) had an error within 5°. Surgeon overestimation and underestimation >5° was found in 1 hip (3.2%) and 2 hips (6.4%) respectively.

Two reasons might explain these precise outcomes. The first explanation is the accuracy and precision of a digital protractor with +/- 0.2° of error guaranteed by the manufacturer. Powerful built-in magnets on its base secured the attachment to the iron surface of the stem handle. It showed the real-time degree of stem anteversion relative to the floor when the leg was held in vertical position. This device was more user-friendly than a manual goniometer which required approximation of one arm parallel with the lower-leg axis and the other arm parallel with the trial-stem axis. Hirata et al [5] used a manual goniometer to estimate the intraoperative stem anteversion in cementless THA. They reported an average error of 7.3° and error within 5° was found in 61% of 73 hips. Likewise, Lee et al [6] found the mean absolute value of discrepancy of 4.5° and the discrepancy was <5° in 48 hips (72%). Whereas estimated stem anteversion by digital protractor alone in the current study had an average error of 1.2° and estimation error within 5° was found in 75% of hips.

Secondly, previous literature has shown that the medial 1/3 of the tibial tubercle was an average of 4 mm lateral to the anteroposterior axis of the tibial component during total knee arthroplasty [15], whereas the intermalleolar midpoint was an average of 4.5 mm lateral to the center of the ankle [16]. The aluminium pipe that was connected between these two points of the leg in this study should represent the mechanical axis of tibia. While the knee was flexed and the femur was internally rotated to position the tibia perpendicularly to the floor, the medial joint space should be widened due to the stretching of the medial collateral ligament. This phenomenon might reduce the constitutional varus alignment of the tibial articular surface and its mechanical axis became perpendicular to the posterior condylar axis of the femur. While the leg was held upright and the bubble of the spirit level was centered, the posterior condylar axis should be parallel with the floor. This assumption was confirmed by Hirata et al [5] who found that the degree of surgeon error for intraoperative estimation of stem anteversion was significantly influenced by the grade of knee osteoarthritis. Surgeons tended to overestimate in the valgus knee and underestimate in the varus knee. Similarly, our study found that the angle overestimation was significantly influenced by the tibio-femoral angle. Hips with angle overestimation >5° had the mean tibio-femoral angle significantly higher, or more valgus, than those with an estimation error within 5°.

There are some limitations to the present study. Firstly, we investigated only on the posterolateral approach in which the leg was turned upright to easily see the centralized-bubble position of the spirit level at the ankle. If the patient was operated in the supine position, surgeons may visualize the femur in a different plane. Likewise, the precision of measurement might differ if we use the direct lateral approach in lateral decubitus, although a previous study found no significant difference in the error measurements for femoral component version by visual estimation when the posterior and modified Hardinge approaches were compared [2]. Secondly, the tibio-femoral angles of the patents in our study were slightly valgus. The precision of this method might not be extrapolated to those with severe knee deformities. Finally, all THAs were performed by the same experienced surgeon. The precision of surgeon estimations can vary from surgeon to surgeon and might be different from those of the surgeon in this study. However, Meermans et al [7] found no difference in the number of safe zone outliers for acetabular component inclination between different surgeons with the use of a digital protractor. To the best of our knowledge, this is the first clinical study that confirms the benefits of a digital protractor and a spirit level to assess the intraoperative anteversion of the femoral component during cementless THA.

The advantages of this novel technique are the high accuracy rates to provide the intra-operative information of stem anteversion which can be used with different stem handles. The surgeon can adjust the anteversion more accurately to achieve the target angle during surgery in a non-invasive, low-cost and time-efficient way. It can reduce the number of anteversion outliers in surgeons with different volumes of practice. Nevertheless, its disadvantages seem to be the prepared invention of a rod connecting between the tibial tubercle and the inter-malleolar midpoint, and its attachment mechanism with the leg and a spirit level. We found that the cable ties and pipe clips were suitable for this purpose. Additionally, the surgical exposure and soft tissue release must be adequate in order to easily hold the leg in the vertical position during the angle estimation. This technique may not be appropriate with surgeons who prefer minimally invasive surgeries.

## Conclusion

Utilization of a digital protractor and a spirit level could provide an accurate estimation of stem anteversion in cementless THA. This technique can determine the intraoperative anteversion with a high precision and can be used with different stem handles in posterolateral approach.

## Data Availability

The datasets used and analyzed in the study are available on request to the corresponding author.

## Abbreviations

THA: Total hip arthroplasty
EKG: Electrocardiogram
ICC: Intra-class correlation coefficients
CT: Computed tomography
CI: Confidence interval
SD: Standard deviation
kg/m^2^: Kilogram per square meter
